# Clinical Frailty Scale and the FRAIL checklist: Can they complement each other?

**DOI:** 10.1186/s13054-023-04451-4

**Published:** 2023-05-04

**Authors:** Emmanuel Hei-Lok Cheung, Jonathan Chun-Hei Cheung, Yu-Yeung Yip

**Affiliations:** 1 Intensive Care Unit, 2/F, North District Hospital, 9 Po Kin Road, Sheung Shui, Hong Kong SAR China; 2 Department of Anaesthesia and Intensive Care, 4/F, Main Clinical Block and Trauma Centre, Prince of Wales Hospital, Sha Tin, Hong Kong SAR China

Dear Editor,

We are grateful to Wernly et al. and the COVIP investigators [1] for their work in evaluating the clinical usefulness of the FRAIL checklist that we developed 2 years ago [2] as a guide to initiate early discussions regarding time-limited trials (TLTs) of intensive care unit (ICU) interventions when necessary.

We recognise the authors' conclusion that the Clinical Frailty Scale (CFS) is associated with 3-month mortality among elderly ICU patients with COVID-19 after adjusting for age, gender, SOFA score, and the decision to withdraw/withhold treatment during the ICU stay, while the FRAIL checklist is not. Interestingly, among the 320 patients reported by Wernly et al., there were more subjects with CFS > 4 (corresponding to requiring assistance with instrumental activities of daily living (IADL) [3]) than those with FRAIL > 0. This observation is surprising since the "F" (Functional impairment) component of the FRAIL checklist is intended to encompass patients who cannot independently perform their IADL [2]. The discrepancy may be due to the FRAIL checklist's items being more loosely defined than the CFS, which could result in subjective interpretations by clinicians. Consequently, we wonder if combining the two measures, using the CFS as an objective assessment of the "F" component, would enhance their predictive power for mortality.

The primary goal of the FRAIL checklist was to identify critically ill patients who may have limited life expectancy or physiological reserve at the initial encounter when prognostic uncertainties are often overwhelming. We believe that initiating discussions about TLTs early on acknowledges these uncertainties and prevents invasive ICU interventions from being extended until the appropriateness of treatment can be more accurately evaluated [4]. Thus, we are curious whether the decision to withhold or withdraw treatment differs significantly between the CFS > 4 and FRAIL > 0 groups. As patients who have life-sustaining treatments withdrawn or withheld during their ICU stay may benefit more from early TLT discussions, this information would help clinicians identify the most suitable group of patients for early TLT discussion.

Lastly, we agree with Wernly et al. that an evidence-based tool such as the CFS should be employed to assess frailty in elderly patients, a practice we also advocate in our clinical work. It may be useful for future research on ICU outcomes to include post-discharge functional and quality of life measurements in addition to mortality for a more holistic patient-centred assessment, as these were not evaluated in the original COVIP study [5].

## Data Availability

Not applicable.

## Abbreviations

TLTs: Time-limited trials
ICU: Intensive care unit
CFS: Clinical Frailty Scale
IADL: Instrumental activities of daily living
